# Supplementary material for: Discovery and application of insertion-deletion (INDEL) polymorphisms for QTL mapping of early life-history traits in Atlantic salmon
Source: BMC Genomics. 2010 Mar 8;11:156. doi: 10.1186/1471-2164-11-156 (PMC2838853; doi:10.1186/1471-2164-11-156)
Supplement: Additional file 2 — Information on developed 76 locus single-run INDEL panel in Atlantic salmon. Information on fluorescence labeling, primer concentrations, PCR pooling and links to alignments, INDEL motifs and GENESCAN (Burge and Karlin 1997) predictions of genes/exons are available in html format. [file 1471-2164-11-156-S2.ZIP › Additionalfile2/table11971.html]

Contig Alignment

```
Base	A B C D E F G H I J K L M N   
1	. . . . . . . . G . . . . G    
2	. . . . . . . . G . . G G G    
3	. . . . . . . . G . . G G G    
4	. . . . . . . . G . . G G G    
5	. . . . . . . . G . . G G G    
6	. . . . . . . . G . . G G G    
7	. . . . . . . . A . . A A A    
8	. . . . . . . . C . . C C C    
9	. . . . . . . . A . . A A A    
10	. . . . . . . . C . . C C C    
11	. . . . . . . . T . . T T T    
12	. . . . . . . . G . . G G G    
13	. . . . . . . . A . . A A A    
14	. . . . . . . . A . . A A A    
15	. . . . . . . . A A A A A A    
16	. . . . . . . . G G G G G G    
17	. . . . . . . . A A A A A A    
18	. . . . . . . . G G G G G G    
19	. . . . . . . . C C C C C C    
20	. . . . . . . . G G G G G G    
21	. . . . . . . . A A A A A A    
22	. . . . . . . . G G G G G G    
23	. . . . . . . . A A A A A A    
24	. . . . . . . . C C C C C C    
25	G . . . . . . G G G G G G G    
26	G . . . . . . G G G G G G G    
27	G . . . . . . G G G G G G G    
28	G . . . . . . G G G G G G G    
29	C . . . . . . C C C C C C C    
30	A . . . . . . A A A A A A A    
31	G . . . . . . G G G G G G G    
32	C . . . . . . C C C C C C C    
33	C . . . . . . C C C C C C C    
34	T . . . . . . T T T T T T T    
35	A . . A . . A A A A A A A A    
36	G . . G . . G G G G G G G G    
37	T . . T . . T T T T T T T T    
38	T . . T . . T T T T T T T T    
39	T . . T . . T T T T T T T T    
40	A . . A . . A A A A A A A A    
41	C . . C . . C C C C C C C C    
42	A . . A . . A A A A A A A A    
43	A . . A . . A A A A A A A A    
44	C . . C . . C C C C C C C C    
45	A . . A A A A A A A A A A A    
46	A . . A A A A A A A A A A A    
47	G . . G G G G G G G G G G G    
48	A . . A A A A A A A A A A A    
49	G . . G G G G G G G G G G G    
50	G . . G G G G G G G G G G G    
51	A . . A A A A A A A A A A A    
52	A . . A A A A A A A A A A A    
53	C . . C C C C C C C C C C C    
54	A . . A A A A A A A A A A A    
55	G . . G G G G G G G G G G G    
56	G . . G G G G G G G G G G G    
57	G . . G G G G G G G G G G G    
58	A . . A A A A A A A A A A A    
59	G . . G G G G G G G G G G G    
60	G . . G G G G G G G G G G G    
61	G . . G G G G G G G G G G G    
62	A . . A A A A A A A A A A A    
63	A . . A A A A A A A A A A A    
64	A . . A A A A A A A A A A A    
65	A . . A A A A A A A A A A A    
66	A . . A A A A A A A A A A A    
67	G . . G G G G G G G G G G G    
68	A . . A A A A A A A A A A A    
69	A . . A A A A A A A A A A A    
70	A . . A A A A A A A A A A A    
71	G . . G G G G G G G G G G G    
72	G . . G G G G G G G G G G G    
73	G . . G G G G G G G G G G G    
74	C . . C C C C C C C C C C C    
75	C . . C C C C C C C C C C C    
76	A . . A A A A A A A A A A A    
77	G . . G G G G G G G G G G G    
78	A . . A A A A A A A A A A A    
79	C . . C C C C C C C C C C C    
80	G . . G G G G G G G G G G G    
81	A . . A A A A A A A A A A A    
82	A . . A A A A A A A A A A A    
83	G . . G G G G G G G G G G G    
84	C . . C C C C C C C C C C C    
85	C . . C C C C C C C C C C C    
86	A . . A A A A A A A A A A A    
87	A . . A A A A A A A A A A A    
88	A . . A A A A A A A A A A A    
89	G . . G G G G G G G G G G G    
90	A . . A A A A A A A A A A A    
91	G . . G G G G G G G G G G G    
92	G . . G G G G G G G G G G G    
93	A . . A A A A A A A A A A A    
94	G . . G G G G G G G G G G G    
95	A . . A A A A A A A A A A A    
96	A . . A A A A A A A A A A A    
97	A . . A A A A A A A A A A A    
98	T . . T T T T T T T T T T T    
99	C . . C C C C C C C C C C C    
100	G . . G G G G G G G G G G G    
101	T . . T T T T T T T T T T T    
102	G . . G G G G G G G G G G G    
103	G . . G G G G G G G G G G G    
104	G . . G G G G G G G G G G G    
105	G . . G G G G G G G G G G G    
106	A . . A A A A A A A A A A A    
107	A . . A A A A A A A A A A A    
108	G . . G G G G G G G G G G G    
109	A . . A A A A A A A A A A A    
110	G G G G G G G G G G G G G G    
111	A A A A A A A A A A A A A A    
112	G G G G G G G G G G G G G G    
113	A A A A A A A A A A A A A A    
114	T T T T T T T T T T T T T T    
115	G G G G G G G G G G G G G G    
116	A A A A A A A A A A A A A A    
117	G G G G G G G G G G G G G G    
118	C C C C C C C C C C C C C C    
119	T T T T T T T T T T T T T T    
120	A A A A A A A A A A A A A A    
121	A A A A A A A A A A A A A A    
122	A A A A A A A A A A A A A A    
123	G G G G G G G G G G G G G G    
124	T T T T T T T T T T T T T T    
125	G G G G G G G G G G G G G G    
126	A A A A A A A A A A A A A A    
127	G G G G G G G G G G G G G G    
128	G G G G G G G G G G G G G G    
129	A A A A A A A A A A A A A A    
130	G G G G G G G G G G G G G G    
131	A A A A A A A A A A A A A A    
132	T T T T T T T T T T T T T T    
133	G G G G G G G G G G G G G G    
134	T T T T T T T T T T T T T T    
135	T T T T T T T T T T T T T T    
136	A A A A A A A A A A A A A A    
137	T T T T T T T T T T T T T T    
138	G G G G G G G G G G G G G G    
139	A A A A A A A A A A A A A A    
140	T T T T T T T T T T T T T T    
141	A A A A A A A A A A A A A A    
142	C C C C C C C C C C C C C C    
143	G G G G G G G G G G G G G G    
144	A A A A A A A A A A A A A A    
145	T T T T T T T T T T T T T T    
146	A A A A A A A A A A A A A A    
147	A A A A A A A A A A A A A A    
148	C C C C C C C C C C C C C C    
149	T T T T T T T T T T T T T T    
150	T T T T T T T T T T T T T T    
151	T T T T T T T T T T T T T T    
152	T T T T T T T T T T T T T T    
153	C C C C C C C C C C C C C C    
154	T T T T T T T T T T T T T T    
155	A A A A A A A A A A A A A A    
156	C C C C C C C C C C C C C C    
157	T T T T T T T T T T T T T T    
158	A A A A A A A A A A A A A A    
159	C C C C C C C C C C C C C C    
160	A A A A A A A A A A A A A A    
161	A A A A A A A A A A A A A A    
162	A A A A A A A A A A A A A A    
163	T T T T T T T T T T T T T T    
164	A A A A A A A A A A A A A A    
165	C C C C C C C C C C C C C C    
166	C C C C C C C C C C C C C C    
167	T T T T T T T T T T T T T T    
168	G G G G G G G G G G G G G G    
169	C C C C C C C C C C C C C C    
170	C C C C C C C C C C C C C C    
171	G G G G G G G G G G G G G G    
172	A A A A A A A A A A A A A A    
173	G G G G G G G G G G G G G G    
174	T T T T T T T T T T T T T T    
175	T T T T T T T T T T T T T T    
176	G G G G G G G G G G G G G G    
177	A A A A A A A A A A A A A A    
178	C C C C C C C C C C C C C C    
179	G G G G G G G G G G G G G G    
180	G G G G G G G G G G G G G G    
181	A A A A A A A A A A A A A A    
182	C C C C C C C C C C C C C C    
183	A A A A A A A A A A A A A A    
184	G G G G G G G G G G G G G G    
185	G G G G G G G G G G G G G G    
186	A A A A A A A A A A A A A A    
187	G G G G G G G G G G G G G G    
188	T T T T T T T T T T T T T T    
189	G G G G G G G G G G G G G G    
190	G G G G G G G G G G G G G G    
191	G G G G G G G G G G G G G G    
192	C C C C C C C C C C C C C C    
193	G G G G G G G G G G G G G G    
194	C C C C C C C C C C C C C C    
195	G G G G G G G G G G G G G G    
196	C C C C C C C C C C C C C C    
197	G G G G G G G G G G G G G G    
198	C C C C C C C C C C C C C C    
199	G G G G G G G G G G G G G G    
200	A A A A A A A A A A A A A A    
201	A A A A A A A A A A A A A A    
202	G G G G G G G G G G G G G G    
203	T T T T T T T T T T T T T T    
204	G G G G G G G G G G G G G G    
205	A A A A A A A A A A A A A A    
206	T T T T T T T T T T T T T T    
207	C C C C C C C C C C C C C C    
208	T T T T T T T T T T T T T T    
209	G G G G G G G G G G G G G G    
210	C C C C C C C C C C C C C C    
211	T T T T T T T T T T T T T T    
212	G G G G G G G G G G G G G G    
213	C C C C C C C C C C C C C C    
214	A A A A A A A A A A A A A A    
215	G G G G G G G G G G G G G G    
216	G G G G G G G G G G G G G G    
217	C C C C C C C C C C C C C C    
218	G G G G G G G G G G G G G G    
219	G G G G G G G G G G G G G G    
220	A A A A A A A A A A A A A A    
221	G G G G G G G G G G G G G G    
222	A A A A A A A A A A A A A A    
223	T T T T T T T T T T T T T T    
224	C C C C C C C C C C C C C C    
225	G G G G G G G G G G G G G G    
226	C C C C C C C C C C C C C C    
227	G G G G G G G G G G G G G G    
228	C C C C C C C C C C C C C C    
229	C C C C C C C C C C C C C C    
230	G G G G G G G G G G G G G G    
231	G G G G G G G G G G G G G G    
232	A A A A A A A A A A A A A A    
233	C C C C C C C C C C C C C C    
234	C C C C C C C C C C C C C C    
235	T T T T T T T T T T T T T T    
236	C C C C C C C C C C C C C C    
237	G G G G G G G G G G G G G G    
238	A A A A A A A A A A A A A A    
239	G G G G G G G G G G G G G G    
240	C C C C C C C C C C C C C C    
241	T T T T T T T T T T T T T T    
242	G G G G G G G G G G G G G G    
243	C C C C C C C C C C C C C C    
244	T T T T T T T T T T T T T T    
245	A A A A A A A A A A A A A A    
246	T T T T T T T T T T T T T T    
247	T T T T T T T T T T T T T T    
248	T T T T T T T T T T T T T T    
249	C C C C C C C C C C C C C C    
250	T T T T T T T T T T T T T T    
251	A A A A A A A A A A A A A A    
252	T T T T T T T T T T T T T T    
253	A A A A A A A A A A A A A A    
254	A A A A A A A A A A A A A A    
255	A A A A A A A A A A A A A A    
256	G G G G G G G G G G G G G G    
257	C C C C C C C C C C C C C C    
258	C C C C C C C C C C C C C C    
259	C C C C C C C C C C C C C C    
260	C C C C C C C C C C C C C C    
261	C C C C C C C C C C C C C C    
262	G G G G G G G G G G G G G G    
263	A A A A A A A A A A A A A A    
264	T T T T T T T T T T T T T T    
265	T T T T T T T T T T T T T T    
266	C C C C C C C C C C C C C C    
267	A A A A A A A A A A A A A A    
268	A A A A A A A A A A A A A A    
269	C C C C C C C C C C C C C C    
270	G G G G G G G G G G G G G G    
271	G G G G G G G G G G G G G G    
272	A A A A A A A A A A A A A A    
273	T T T T T T T T T T T T T T    
274	C C C C C C C C C C C C C C    
275	T T T T T T T T T T T T T T    
276	G G G G G G G G G G G G G G    
277	T T T T T T T T T T T T T T    
278	G G G G G G G G G G G G G G    
279	T T T T T T T T T T T T T T    
280	C C C C C C C C C C C C C C    
281	A A A A A A A A A A A A A A    
282	G G G G G G G G G G G G G G    
283	A A A A A A A A A A A A A A    
284	G G G G G G G G G G G G G G    
285	C C C C C C C C C C C C C C    
286	A A A A A A A A A A A A A A    
287	T T T T T T T T T T T T T T    
288	C C C C C C C C C C C C C C    
289	A A A A A A A A A A A A A A    
290	C C C C C C C C C C C C C C    
291	C C C C C C C C C C C C C C    
292	G G G G G G G G G G G G G G    
293	G G G G G G G G G G G G G G    
294	C C C C C C C C C C C C C C    
295	T C C T T T T T T T T T T T    
296	G G G G G G G G G G G G G G    
297	G G G G G G G G G G G G G G    
298	C C C C C C C C C C C C C C    
299	C C C C C C C C C C C C C C    
300	T T T T T T T T T T T T T T    
301	C C C C C C C C C C C C C C    
302	T T T T T T T T T T T T T T    
303	C C C C C C C C C C C C C C    
304	G G G G G G G G G G G G G G    
305	C C C C C C C C C C C C C C    
306	T T T T T T T T T T T T T T    
307	T T T T T T T T T T T T T T    
308	G G G G G G G G G G G G G G    
309	G G G G G G G G G G G G G G    
310	G G G G G G G G G G G G G G    
311	T T T T T T T T T T T T T T    
312	T T T T T T T T T T T T T T    
313	T T T T T T T T T T T T T T    
314	C C C C C C C C C C C C C C    
315	C C C C C C C C C C C C C C    
316	T T T T T T T T T T T T T T    
317	G G G G G G G G G G G G G G    
318	C C C C C C C C C C C C C C    
319	C C C C C C C C C C C C C C    
320	T T T T T T T T T T T T T T    
321	T T T T T T T T T T T T T T    
322	T T T T T T T T T T T T T T    
323	G G G G G G G G G G G G G G    
324	A A A A A A A A A A A A A A    
325	G G G G G G G G G G G G G G    
326	C C C C C C C C C C C C C C    
327	T T T T T T T T T T T T T T    
328	C C C C C C C C C C C C C C    
329	A A A A A A A A A A A A A A    
330	A A A A A A A A A A A A A A    
331	A A A A A A A A A A A A A A    
332	C C C C C C C C C C C C C C    
333	C C C C C C C C C C C C C C    
334	T T T T T T T T T T T T T T    
335	G G G G G G G G G G G G G G    
336	T T T T T T T T T T T T T T    
337	C C C C C C C C C C C C C C    
338	G G G G G G G G G G G G G G    
339	T T T T T T T T T T T T T T    
340	T T T T T T T T T T T T T T    
341	T T T T T T T T T T T T T T    
342	T T T T T T T T T T T T T T    
343	C C C C C C C C C C C C C C    
344	T T T T T T T T T T T T T T    
345	T T T T T T T T T T T T T T    
346	T T T T T T T T T T T T T T    
347	G G G G G G G G G G G G G G    
348	G G G G G G G G G G G G G G    
349	T T T T T T T T T T T T T T    
350	C C C C C C C C C C C C C C    
351	C C C C C C C C C C C C C C    
352	A A A A A A A A A A A A A A    
353	A A A A A A A A A A A A A A    
354	G G G G G G G G G G G G G G    
355	G G G G G G G G G G G G G G    
356	G G G G G G G G G G G G G G    
357	G G G G G G G G G G G G G G    
358	A A A A A A A A A A A A A A    
359	G G G G G G G G G G G G G G    
360	C C C C C C C C C C C C C C    
361	A A A A A A A A A A A A A A    
362	G G G G G G G G G G G G G G    
363	C C C C C C C C C C C C C C    
364	A A A A A A A A A A A A A A    
365	A A A A A A A A A A A A A A    
366	C C C C C C C C C C C C C C    
367	C C C C C C C C C C C C C C    
368	G A A A A A A G A A A A A A    
369	A A A A A A A A A A A A A A    
370	C C C C C C C C C C C C C C    
371	A A A A A A A A A A A A A A    
372	C C C C C C C C C C C C C C    
373	A A A A A A A A A A A A A A    
374	G G G G G G G G G G G G G G    
375	A A A A A A A A A A A A A A    
376	A A A A A A A A A A A A A A    
377	C C C C C C C C C C C C C C    
378	A A A A A A A A A A A A A A    
379	G G G G G G G G G G G G G G    
380	T T T T T T T T T T T T T T    
381	G G G G G G G G G G G G G G    
382	C C C C C C C C C C C C C C    
383	T T T T T T T T T T T T T T    
384	G G G G G G G G G G G G G G    
385	T T T T T T T T T T T T T T    
386	G G G G G G G G G G G G G G    
387	G G G G G G G G G G G G G G    
388	T T T T T T T T T T T T T T    
389	C C C C C C C C C C C C C C    
390	T T T T T T T T T T T T T T    
391	C C C C C C C C C C C C C C    
392	G G G G G G G G G G G G G G    
393	C C C C C C C C C C C C C C    
394	C C C C C C C C C C C C C C    
395	C C C C C C C C C C C C C C    
396	C C C C C C C C C C C C C C    
397	A A A A A A A A A A A A A A    
398	C C C C C C C C C C C C C C    
399	C C C C C C C C C C C C C C    
400	T T T T T T T T T T T T T T    
401	T T T T T T T T T T T T T T    
402	C C C C C C C C C C C C C C    
403	T T T T T T T T T T T T T T    
404	G G G G G G G G G G G G G G    
405	T T T T T T T T T T T T T T    
406	C C C C C C C C C C C C C C    
407	A A A A A A A A A A A A A A    
408	T T T T T T T T T T T T T T    
409	T T T T T T T T T T T T T T    
410	G G G G G G G G G G G G G G    
411	G G G G G G G G G G G G G G    
412	G G G G G G G G G G G G G G    
413	G G G G G G G G G G G G G G    
414	T T T T T T T T T T T T T T    
415	C C C C C C C C C C C C C C    
416	C C C C C C C C C C C C C C    
417	G G G G G G G G G G G G G G    
418	A A A A A A A A A A A A A A    
419	G G G G G G G G G G G G G G    
420	T T T T T T T T T T T T T T    
421	T T T T T T T T T T T T T T    
422	A A A A A A A A A A A A A A    
423	T T T T T T T T T T T T T T    
424	C C C C C C C C C C C C C C    
425	A A A A A A A A A A A A A A    
426	C C C C C C C C C C C C C C    
427	C C C C C C C C C C C C C C    
428	A A A A A A A A A A A A A A    
429	T T T T T T T T T T T T T T    
430	C C C C C C C C C C C C C C    
431	G G G G G G G G G G G G G G    
432	C C C C C C C C C C C C C C    
433	T T T T T T T T T T T C C T    
434	G G G G G G G G G G G G G G    
435	C C C C C C C C C C C C C C    
436	T T T T T T T T T T T T T T    
437	G G G G G G G G G G G G G G    
438	C C C C C C C C C C C C C C    
439	T T T T T T T T T T T T T T    
440	C C C C C C C C C C C C C C    
441	A A A A A A A A A A A A A A    
442	T T T T T T T T T T T T T T    
443	G G G G G G G G G G G G G G    
444	A A A A A A A A A A A A A A    
445	C C C C C C C C C C C C C C    
446	T T T T T T T T T T T T T T    
447	G G G G G G G G G G G G G G    
448	C C C C C C C C C C C C C C    
449	G G G G G G G G G G G G G G    
450	T T T T T T T T T T T T T T    
451	T T T T T T T T T T T T T T    
452	G G G G G G G G G G G G G G    
453	G G G G G G G G G G G G G G    
454	A A A A A A A A A A A A A A    
455	G G G G G G G G G G G G G G    
456	T T T T T T T T T T T T T T    
457	G G G G G G G G G G G G G G    
458	A A A A A A A A A A A A A A    
459	A A A A A A A A A A A A A A    
460	A A A A A A A A A A A A A A    
461	T T T T T T T T T T T T T T    
462	A A A A A A A A A A A A A A    
463	C C C C C C C C C C C C C C    
464	T T T T T T T T T T T T T T    
465	T T T T T T T T T T T T T T    
466	C C C C C C C C C C C C C C    
467	T T T T T T T T T T T T T T    
468	G G G G G G G G G G G G G G    
469	C C C C C C C C C C C C C C    
470	C C C C C C C C C C C C C C    
471	A A A A A A A A A A A A A A    
472	A A A A A A A A A A A A A A    
473	C C C C C C C C C C C C C C    
474	T T T T T T T T T T T T T T    
475	G G G G G G G G G G G G G G    
476	T T T T T T T T T T T T T T    
477	A A A A A A A A A A A A A A    
478	T T T T T T T T T T T T T T    
479	T T T T T T T T T T T T T T    
480	T T T T T T T T T T T T T T    
481	T T T T T T T T T T T T T T    
482	C C C C C C C C C C C C C C    
483	A A A A A A A A A A A A A A    
484	G G G G G G G G G G G G G G    
485	T T T T T T T T T T T T T T    
486	C C C C C C C C C C C C C C    
487	T T T T T T T T T T T T T T    
488	A A A A A A A A A A A A A A    
489	T T T T T T T T T T T T T T    
490	T T T T T T T T T T T T T T    
491	A A A A A A A A A A A A A A    
492	C C C C C C C C C C C C C C    
493	C C C C C C C C C C C C C C    
494	T T T T T T T T T T T T T T    
495	T T T T T T T T T T T T T T    
496	T T T T T T T T T T T T T T    
497	T T T T T T T T T T T T T T    
498	T T T T T T T T T T T T T T    
499	C G G G G G G G G G G G G G    
500	. A A A A A A A A A A A A A    
501	. G G G G G G G G G G G G G    
502	. A A A A A A A A A A A A A    
503	. C C C C C C C C C C C C C    
504	. G G G G G G G G G G G G G    
505	. T T T T T T T T T T T T T    
506	. T T T T T T T T T T T T T    
507	. G G G G G G G G G G G G G    
508	. A A G A A G A A A A A A A    
509	. T T T T T T T T T T T T T    
510	. A A A A A A A A A A A A A    
511	. G G G G G G G G G G G G G    
512	. A A A A A A A A A A A A A    
513	. T T T T T T T T T T T T T    
514	. A A A A A A A A A A A A A    
515	. C C C C C C C C C C C C C    
516	. G G G G G G G G G G G G G    
517	. A A A A A A A A A A A A A    
518	. G G G G G G G G G G G G G    
519	. G G G G G G G G G G G G G    
520	. C C C C C C C C C C C C C    
521	. A A A A A A A A A A A A A    
522	. G G G G G G G G G G G G G    
523	. G G G G G G G G G G G G G    
524	. G G G G G G G G G G G G G    
525	. G G G G G G G G G G G G G    
526	. T T T T T T T T T T T T T    
527	. G G G G G G G G G G G G G    
528	. T T T T T T T T T T T T T    
529	. G G G G G G G G G G G G G    
530	. T T T T T T T T T T T T T    
531	. G G G G G G G G G G G G G    
532	. T T T T T T T T T T T T T    
533	. G G G G G G G G G G G G G    
534	. G G G G G G G G G G G G G    
535	. G G G G G G G G G G G G G    
536	. A A A A A A A A A A A A A    
537	. G G G G G G G - - - - - -  6	 indel  
538	. A A A A A A A - - - - - -  6	 indel  
539	. G G G G G G G G G G G G G    
540	. C C C C C C C C C C C C C    
541	. C C C C C C C C C C C C C    
542	. G G G G G G G A A A A A A  6	 transition  
543	. A A A A A A A A A A A A A    
544	. C C C C C C C C C C C C C    
545	. T T T T T T T T T T T T T    
546	. G G G G G G G G G G G G G    
547	. A A A A A A A A A A A A A    
548	. G G G G G G G G G G G G G    
549	. G G G G G G G G G G G G G    
550	. C C C C C C C C C C C C C    
551	. C C C C C C C C C C C C C    
552	. A A A A A A A A A A A A A    
553	. A A A A A A A A A A A A A    
554	. G G G G G G G G G G G G G    
555	. G G G G G G G G G G G G G    
556	. T T T T T T T T T T T T T    
557	. G G G G G G G G G G G G G    
558	. G G G G G G G G G G G G G    
559	. T T T T T T T T T T T T T    
560	. G G G G G G G G G G G G G    
561	. T T T T T T T T T T T T T    
562	. T T T T T T T T T T T T T    
563	. T T T T T T T T T T T T T    
564	. A A A A A A A A A A A A A    
565	. A A A A A A A A A A A A A    
566	. A A A A A A A A A A A A A    
567	. A A A A A A A A A A A A A    
568	. A A A A A A A A A A A A A    
569	. A A A A A A A A A A A A A    
570	. A A A A A A A A A A A A A    
571	. T T T T T T T T T T T T T    
572	. A A A A A A A A A A A A A    
573	. T T T T T T T T T T T T T    
574	. T T T T T T T T T T T T T    
575	. A A A A A A A A A A A A A    
576	. A A A A A A A A A A A A A    
577	. C C C C C C C C C C C C C    
578	. G G G G G G G G G G G G G    
579	. T T T T C T T T T T T T T    
580	. . . . . . . . T T T T T T    
581	. . . . . . . . A A A A A A    
582	. . . . . . . . G G G G G G    
583	. . . . . . . . T T T T T T    
584	. . . . . . . . G G G G G G    
585	. . . . . . . . G G G G G G    
586	. . . . . . . . G G G G G G    
587	. . . . . . . . G G G G G G    
588	. . . . . . . . T T T T T T    
589	. . . . . . . . A A A A A A    
590	. . . . . . . . G G G G G G    
591	. . . . . . . . C C C C C C    
592	. . . . . . . . G G G G G G    
593	. . . . . . . . C C C C C C    
594	. . . . . . . . A A A A A A    
595	. . . . . . . . T T T T T T    
596	. . . . . . . . A A A A A A    
597	. . . . . . . . T T T T T T    
598	. . . . . . . . G G G G G G    
599	. . . . . . . . T T T T T T    
600	. . . . . . . . G G G G G G    
601	. . . . . . . . G G G G G G    
602	. . . . . . . . C C C C C C    
603	. . . . . . . . T T T T T T    
604	. . . . . . . . A A A A A A    
605	. . . . . . . . G G G G G G    
606	. . . . . . . . C C C C C C    
607	. . . . . . . . T T T T T T    
608	. . . . . . . . T T T T T T    
609	. . . . . . . . G G T G G G    
610	. . . . . . . . C C . C C C
```
